# Geriatric rehabilitation in Germany, Austria, and Switzerland (DACH region): a current state analysis

**DOI:** 10.1007/s41999-026-01461-7

**Published:** 2026-03-29

**Authors:** M. Skoumal, M. Honegger, S. Grund, P. Benzinger, S. Bachmann, T. Münzer, S. M. Goetz, T. E. Dorner, B. Iglseder, C. Pertinatsch, B. Neubacher, C. Kadane, S. Lindner-Rabl, R. Roller-Wirnsberger

**Affiliations:** 1 Department of Internal Medicine, Research Unit Services for Old Age and Life Long Health, Medical University of Graz, Graz, Austria; 2 Department for Scientific Research in Rehabilitation, Pension Insurance Austria, Vienna, Austria; 3 Geriatric Center of the University of Heidelberg, Agaplesion Bethanien Hospital, Heidelberg, Germany; 4 Kliniken Valens, Valens, Switzerland; 5 Department of Geriatrics, Inselspital, Universitätsspital Bern, Bern, Switzerland; 6 Geriatrische Klinik St. Gallen, St. Gallen, Switzerland; 7 Spital Thurgau AG, Frauenfeld, Switzerland; 8 Academy of Ageing Research, Haus der Barmherzigkeit, Vienna, Austria; 9 Centre for Public Health, Department of Social and Preventive Medicine, Medical University Vienna, Vienna, Austria; 10 Department of Geriatric Medicine, Christian Doppler University Hospital, Paracelsus Medical University, Salzburg, Austria; 11 Swiss Society of Physical Medicine and Rehabilitation, Geneva, Switzerland

**Keywords:** Geriatric rehabilitation, ICF, Comprehensive geriatric assessment, Integrated care pathway, Older person

## Abstract

**Purpose:**

Geriatric rehabilitation (GR) is a key component of integrated care for older adults. This study aims to outline the current state of geriatric rehabilitation in the DACH region, highlighting national differences, comparing structural frameworks, and assessing the implementation of the European consensus statement and WHO recommendations.

**Methods:**

An online survey was conducted within a working group of GR experts from the three countries, officially nominated by the national geriatric societies. The questionnaire was developed based on a search of recent literature on the GR process and supplemented by desktop research on country-specific care structures based on the European Consensus Statement on GR.

**Results:**

The most significant structural difference relates to Austria, where phase 2 rehabilitation for older patients is offered only in disease-oriented centers, but no service is currently available for specific GR. All three countries use different standardized admission assessments, therapy minutes and country-specific geriatric qualifications. Digital health solutions and mobile/home-based GR are still in development.

**Conclusion:**

The recommendations of the European Consensus Statement are largely adopted, with country variations. In order to ensure the evidence-based long-term effectiveness, quality and sustainability of GR, there is a need for standardized quality criteria and innovative models. Such models will facilitate the identification of best practices grounded in robust evidence and focused on optimizing care. In light of these considerations, further research in this field is needed.

Key Summary Points.

**Aim:**

This article aims to present the current state of GR in the DACH region, in order to detect differences, compare the national structures and to show the extent to which the European Consensus Statement and WHO recommendations have been implemented.

**Findings:**

The most significant structural differences relate to the absence of specific phase 2 GR in Austria, different standardized admission assessments, therapy minutes and country-specific geriatric qualifications. Mobile/home-based GR as well as digital health solutions are still in development.

**Message:**

Future considerations for GR should include the development of standardized quality criteria and the integration of innovative models, particularly in mobile and home-based settings, to ensure long-term effectiveness, evidence-based quality, and sustainability.

**Supplementary Information:**

The online version contains supplementary material available at 10.1007/s41999-026-01461-7.

## Introduction

Worldwide, the need for rehabilitation is expected to rise due to demographic changes, with an increasing number of older people suffering from chronic conditions and multimorbidity, driving demand for tailored geriatric rehabilitation (GR) services [1, 2]. At the same time, the ongoing digital transformation of healthcare sets new opportunities to improve care including older individuals [3, 4].

National healthcare systems are increasingly forced to adapt to these developments, ensuring that older patients with multiple chronic conditions have access to person-centered rehabilitation. Care should be of high quality, coordinated, affordable, accessible, and responsive to the needs and rights of older people [5]. Its purpose is to reduce or overcome limitations in functioning, activity, and participation, as outlined by the biopsychosocial model of the International Classification of Functioning, Disability and Health (ICF) [6, 7].

In this context, GR plays a key role in supporting older people to regain functional abilities and improve their quality of life. The European Consensus Statement on GR [8] defines GR as a multidimensional approach of “diagnostic and therapeutic interventions, the purpose of which is to optimize functional capacity, promote activity and preserve functional reserve and social participation in older people with disabling impairments”. GR, according to this consensus, spans different medical conditions, is delivered by specially trained teams, and is aimed at early, participation-focused rehabilitation for multimorbid patients, based on the ICF framework [8]. The definition of personalized rehabilitation goals related to social participation of the patients, based on the Comprehensive Geriatric Assessment (CGA) and using shared decision-making (SDM) is “the core” of this care model [9, 10]. This approach has been shown to be effective and efficient in supporting sustainable rehabilitation outcomes [11–14].

Similar to heterogenic care structures for GR in Europe [15] and despite shared language and cultural similarities, Germany, Austria, and Switzerland (DACH) differ in several aspects of their healthcare systems, including service structures, policy frameworks, funding models, and clinical approaches, all of which can influence how GR is organized. Understanding these differences is crucial for identifying future innovation capacities, promoting the sharing of best practices and paving the way for the alignment of GR in the DACH region with international evidence-based recommendations.

In this article, we present the current state of GR and compare its structures across the DACH region. Our findings will be evaluated against GR recommendations supported by the European Society of Geriatric Medicine (EuGMS) [8]. This publication represents the first step in a comprehensive research project aimed at achieving consensus on GR in the DACH region focusing on barriers, facilitators and best practices [16].

## Methods

### Survey design

In order to comprehensively assess the current status of GR in the DACH region, members of the project team (MS, MH, CP, RRW) designed and pretested a structured online survey.

**Fig. 1:**
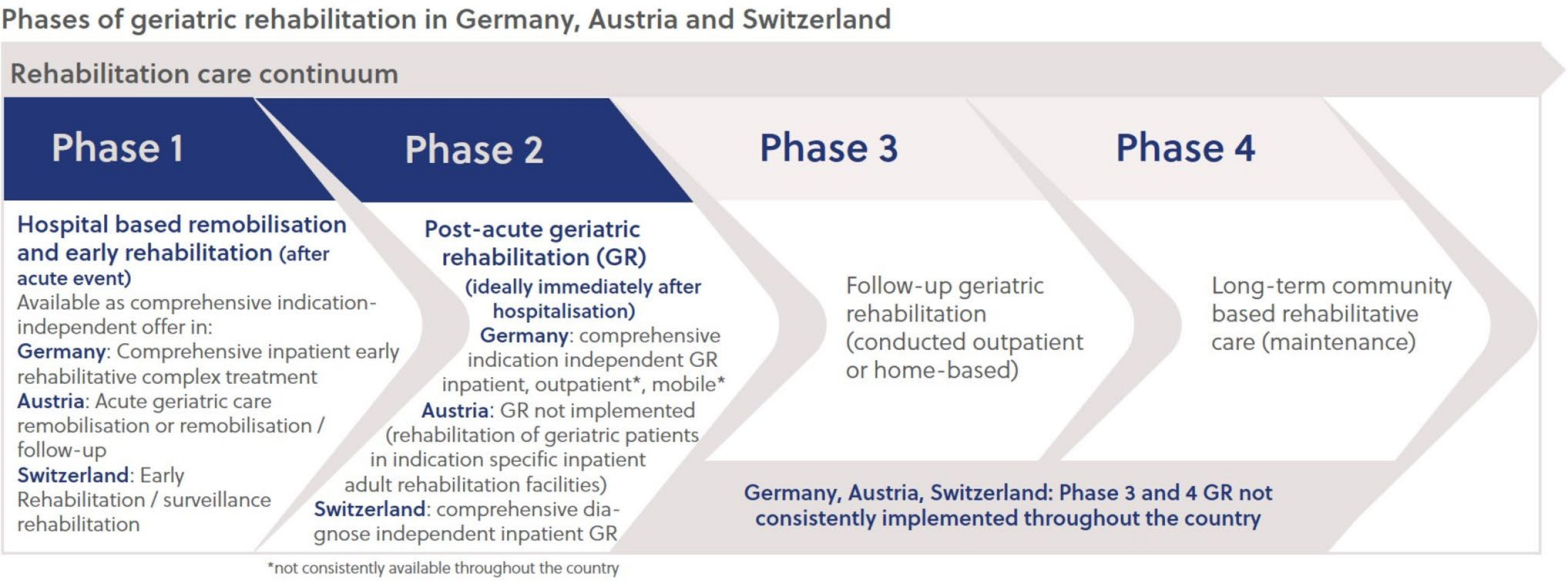
Phases of GR in the DACH region. This figure describes the different phases of GR in Germany, Austria and Switzerland

The questionnaire was developed based on a search of recent literature on the GR process and supplemented by desktop research on country-specific care structures. The topics within the survey were based on the structure of the European Consensus Statement on GR [8] and included the following main subjects: (1) GR care processes according to the WHO rehabilitation phase model (phases 1–4; including guidelines and protocols) (see Fig. 1) [17], (2) resources and structures (e.g. team structure, quality assurance and control systems), (3) digitalization efforts, such as use of eHealth and tele-rehabilitation.

To ensure usability and clarity, the questionnaire was pretested by experts (CK, BN, SLR) with relevant expertise in questionnaire design, rehabilitation structures and research. Experts’ feedback on the primary survey design was included in the final version of the document. The survey was launched online using LimeSurvey™ between 6 February and 16 April 2025, operated in the IT infrastructure of the Medical University of Graz, to ensure data protection, accessibility, and ease of use [18, 19]. The survey questions are available in Additional file 1.

### Stakeholder selection

The survey was conducted within a working group of rehabilitation experts from the three countries, officially nominated in October 2024 by the national geriatric (and gerontological) societies of Germany (German Society for Geriatrics), the Austrian Society for Geriatrics and Gerontology and Switzerland (Swiss Society for Geriatrics).

The inclusion of officially nominated experts from the three national geriatric societies ensured methodological rigor and balanced representation of national perspectives and healthcare contexts.

### Data extraction

Completed questionnaires were reviewed by two independent analysts for completeness and plausibility (MH, BN). To contextualize the data, country-specific discrepancies or missing responses were clarified in three bilateral videoconference meetings between two research team members (MS, MH), and the respective national experts (SG, PB, SB, TM). The discussions aimed to deepen understanding and reach consensus [20]. Consequently, the recaptured and final quantitative data were included into a structured comparative table based on the questionnaire’s thematic framework.

### Data analysis

For comparison of health service delivery structures and the frequency of individual medical procedures across Germany, Switzerland, and Austria, descriptive analysis was applied. Due to the limited number of comparison groups, no inferential statistical tests were applied. For each country, the number of services provided in a specified care setting was summarized, using absolute counts and proportion data. Free-text responses were analyzed using qualitative content analysis following the framework method, structured by predefined research questions. Key statements were extracted, organized, and analyzed for commonalities, differences, and country-specific features [21, 22]. This approach enabled a systematic, comparative interpretation of the results in light of the healthcare realities in Germany, Austria, and Switzerland as described. The overall reporting of findings followed the Standards for Reporting Qualitative Research (SRQR) to ensure a transparent and comprehensible account of methodology, data sources, and interpretive analysis [23].

## Results

### General description

The expert group included six GR specialists, two from each country (four of them geriatricians and two rehabilitation specialists, one female and five males). The members gave informed consent and all are listed as authors of this paper.

In total, the survey consisted of 61 questions, including free-text fields, structured according to the different rehabilitation phases, and resulted in 254 single responses. Experts from Germany and Switzerland were able to answer all detailed questions concerning phases 1 (acute geriatric care/remobilization/early rehabilitation) and 2 (post-acute inpatient/outpatient GR). The Austrian experts, however, could only provide input for phase 1 of the GR process (see later). Questions related to phases 3 (outpatient GR) and 4 (follow-up programs) could not be answered by all three countries.

The definition of a geriatric patient in Germany, Austria, and Switzerland is consistent with the European Consensus Statement definition. It is defined as a patient with multimorbidity typical of older age groups and advanced age (predominantly over 70 years old). Multimorbidity (with the presence of at least two geriatric syndromes, such as frailty, sarcopenia, or delirium—Swiss definition) is considered to take precedence over chronologic age, with an age-related increased risk of complications and secondary diseases, chronicity, and an increased risk of loss of autonomy with deterioration of self-care status (Germany, Austria) [24].

#### Have the rehabilitation phases recommended by the WHO been implemented in all three countries?

The phases of rehabilitation were adopted from the Austrian rehabilitation plan, which is based on the WHO phases of rehabilitation [17, 25]. Phase 1 is the hospital-based remobilization and early rehabilitation after an acute event and phase 2 is post-acute rehabilitation, after hospitalization. Phase 3 refers to the ambulatory care of rehabilitation, which is conducted in the outpatient healthcare setting or home-based. Phase 4 is concerned with long-term community-based rehabilitation and maintenance [17]. Furthermore, geriatric prehabilitation refers to the process of optimizing the physical and mental health of older adults before surgery or medical treatment to improve their recovery and reduce complications [26].

The analysis of expert responses on structures, processes, and clinical guidelines indicates that patients undergoing GR are remobilized or rehabilitated independent from their leading diagnosis/clinical referral cause in all three countries in phase 1 and in Germany and Switzerland also in phase 2. The most significant structural difference addresses Austria, where phase 2 rehabilitation for older patients is offered in disease-oriented rehabilitation centers for eight main leading ICD clusters: orthopedics, neurology, cardiology, gastroenterology, pulmonology, oncology, metabolism (diabetes), and mental illness [27]. There is currently no phase 2 rehabilitation service available in Austria for GR, which may be compared to structures present in the other DACH countries. Consequently, Austrian responses presented in this paper exclusively refer to phase 1 rehabilitation. This phase of GR is generally delivered as acute geriatric care/remobilization or remobilization/follow-up, often integrated into hospitals and focused on orthotraumatological, neurologic, cardiological, or metabolic conditions. Remarkably, phase 1 GR offers in Austria may also be provided in day clinics or through mobile services, though not nationwide [28].

In Germany, phase 1 GR is delivered as a comprehensive inpatient early rehabilitative complex treatment, outpatient, or in day clinics, and in Switzerland as surveillance rehabilitation or early rehabilitation. The term “geriatric rehabilitation” applies to post-acute phase 2 GR in Germany and Switzerland predominantly. In Germany, it is delivered in inpatient, outpatient, mobile services or day clinic settings; in Switzerland, almost exclusively in inpatient settings due to limited availability of outpatient programs. In this context of phase 2 GR, patients receive rehabilitation aligned with the WHO recommendations and with participation-oriented rehabilitation according to the ICF, standard geriatric processes, such as CGA, interdisciplinary teamwork, social integration, and discharge planning.

Notably, nursing homes are not accredited formal GR facilities in all three countries, and geriatric prehabilitation, as well as GR rehabilitation phases 3 and 4, is missing in all DACH countries.

### Are the results of the European consensus statement on GR transferable to the results of the current state analysis of GR in the DACH region?

#### Team structure

**Fig. 2:**
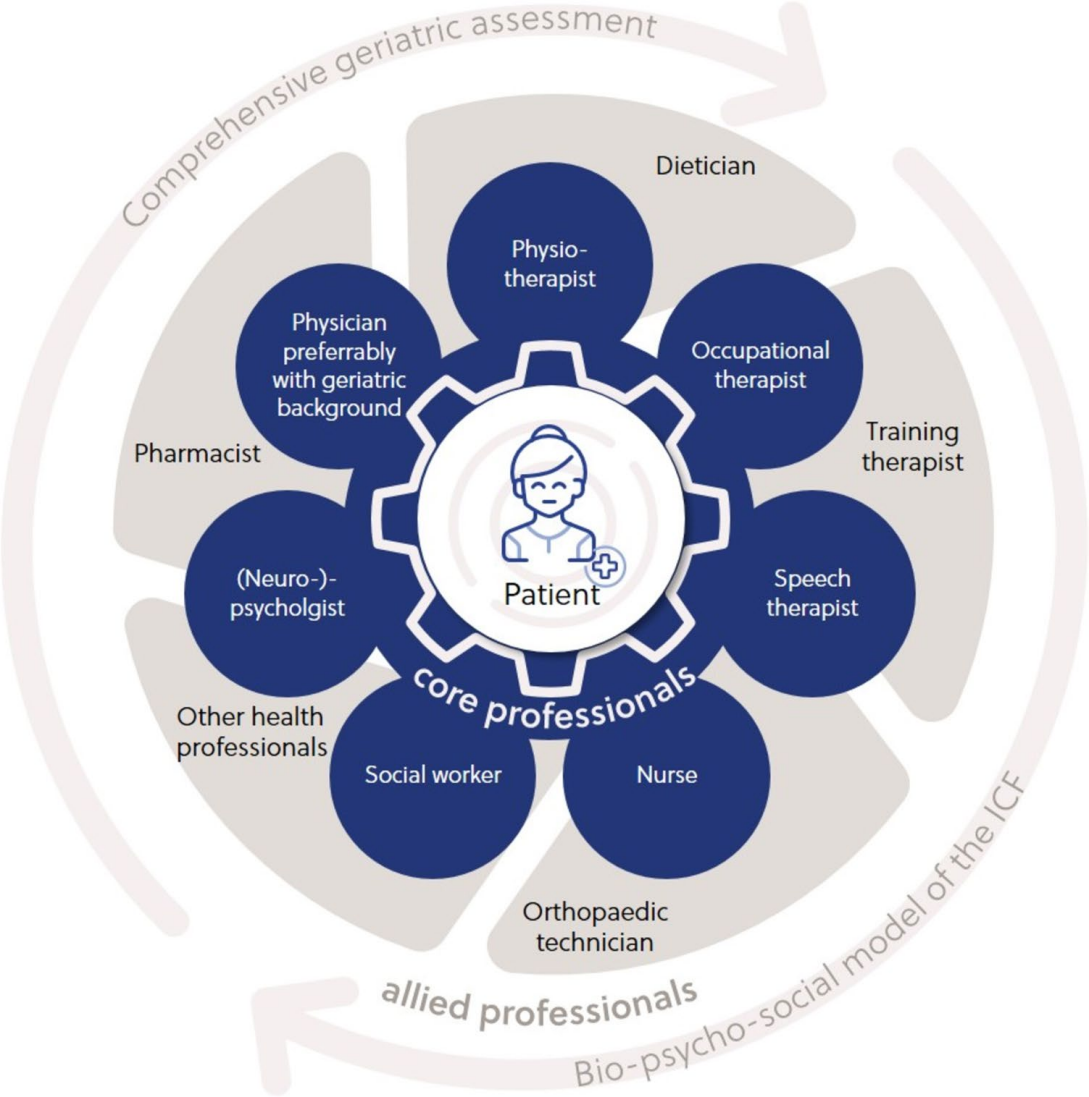
Overview of the multidisciplinary GR team. This figure provides an overview of the multidisciplinary team currently working in GR in the DACH region according to the biopsychosocial model of the ICF and following a CGA. The professional groups are categorized as either mandatory or optional

As recommended by the European Consensus Statement [8], all three countries follow a multidisciplinary team approach. As may be seen from Fig. 2, there are mandatory staffing requirements for the core team for phases 1 and 2 GR physicians with country-specific geriatric qualifications and additional specialists in neurology/psychiatry and orthopedics, physiotherapists, occupational therapists, nurses, psychologists, and social workers in all three countries, and speech therapists in Germany and Switzerland. Additionally, neuropsychological care is delivered only in Germany.

All countries describe the optional availability of capacities, such as dietitians, training therapists, pharmacists, orthopedic technicians, and other health professionals (e.g. massage therapists and medical technical assistants).

Further, the survey asked for barriers and facilitators for human resources development. Similar to the European Consensus Statement on GR [8], the responses for Germany and Switzerland indicate significant overall workforce shortages and increased workloads, particularly in nursing and medical professions. Additional barriers identified across all three countries include a lack of standardized assessment tools, limited bed capacity and inadequate reimbursement models [29, 30]. Regulatory frameworks for GR exist throughout the whole DACH region.

#### Geriatric rehabilitation process

This survey also evaluated access criteria for GR, ICF orientation, and time of therapy for GR. Standardized admission assessments to phase 1 GR are in place in all three countries. Switzerland uses the functional independence measure (FIM) or extended Barthel index (EBI) [31, 32] while Germany and Austria use the Barthel Index [33, 34].

All three countries implemented a participation-oriented model of care based on the ICF. CGA is conducted on admission and discharge in Germany and Switzerland in phases 1 and 2, and at admission to phase 1 in Austria. Rehabilitation goals are jointly defined at the levels of functioning, activity, and participation, involving the patient and, where necessary, their informal caregivers. In Switzerland, the goals set during phase 1 are additionally, but not exclusively, based on the results of the admission assessment.

Furthermore, the survey examined how many minutes of therapy per week are foreseen for GR and whether standardized therapy regimens or individualized therapies are offered. The answers showed varying clinical-therapeutic approaches. Germany uses both standardized and individualized therapy regimens. Austria uses rather “individualized and person-centered regimens”. There were remarkable differences in responses to the number of therapy minutes per week. In Germany, therapy minutes per week are only mandatory for phase 1, at an average of 600 min (ranging from 300 to 900). In Switzerland, such a requirement only applies to phase 2, with a minimum of 300 min. In Austria, there are no prescribed therapy minutes. Interdisciplinary team meetings and discharge planning are mandatory elements in all countries and phases in which GR is offered.

### Results on digital transformation in GR in the DACH region

Due to the possible impact of digital health applications, this survey also asked for current availabilities. The utilization of eHealth approaches in telehealth, telemedicine and telecare is scattered and in its nascent stages. All three countries use these technologies to varying degrees, though not comprehensively. Mobile apps and health sensors are present across the regions. Austria and Switzerland use virtualreality. Austria and Switzerland use virtual reality, Germany and Switzerland recently implemented exergaming in therapeutic plans. Germany reports use of assistive robotics and support technologies. Austria and Switzerland use virtual reality. AI is not yet in use regularly. In Switzerland, phase 2 tele-rehabilitation is under development as a provider-specific, multidisciplinary approach.

## Discussion

It was the major aim of this work to elaborate a status quo on GR in Germany, Austria and Switzerland in order to detect differences between the countries and to have the basis for implementation of the EuGMS recommendations in GR [8]. This work is the first step (maturity assessment) of a cross-regional project in the DACH region, aiming to develop a joint consensus statement and best practices for GR. The maturity assessment is a crucial component as it maps the current situation and highlights the differences between the countries, providing valuable insights into how the GR process is implemented. The authors build their analysis on a structured survey based on the content of the European Consensus Statement [8]. Our work focused on the applicability of the European Consensus Statement of the EuGMS in systems running in the DACH region [2, 8].

As may be seen from Fig. 1, evidence-based GR should be built in four phases according to WHO standards. A major difference between countries is the mode of admittance to phase 1 GR. While the EBI [32], the FIM [31] and the Clinical Frailty Scale [35] are applied in Switzerland, the Barthel Index [34] is used in Germany to decide on admission to GR. The decision regarding admission to GR is not contingent solely on the Barthel Index; rather, it is informed by a series of analogous applications for inclusion. The subject of guidance is addressed in the Austrian Geriatrics Process Manual [28]. Due to the differing admission selection assessment instruments used, there may be major differences in patient criteria and needs among phase 1 rehabilitation patients. This is a major finding as data on interventions and therapeutic offers as well as GR outcomes strongly depend on that possible selection bias. Acknowledging this heterogeneity, rehabilitation offers have to be tailored to the individual, optimizing outcomes by matching patients to the most beneficial interventions and potentially improving resource allocation by avoiding ineffective or overly expensive treatments for certain subgroups; they are a major argument for implementation of the ICF-based concept of GR, even beyond phase 1 rehabilitation offers [36].

This is an interesting finding as the project could demonstrate additional and substantial differences across the DACH countries concerning all other phases of rehabilitation offers for GR: If social participation targeted during goal setting at the beginning of the rehabilitation process is not reached in phase 1 GR, post-acute phase 2 rehabilitation should be available in designated GR centers. This situation is actually not covered in Austria, which is different from Germany and Switzerland. In Austria, geriatric patients have the opportunity to continue their rehabilitation process in disease-oriented rehabilitation centers. Their offers focus on specific acute or chronic health conditions like stroke or cardiovascular disease and do not follow the principles for GR described in the European Consensus Statement [8].

Consequently, older people do not receive diagnosis-independent rehabilitation tailored to their functional, cognitive and social needs, although comprehensive GR has been shown to reduce rehospitalization, long-term care dependency, and loss of independence [14, 37]. Therefore, a more patient-centered or participation-centered approach that considers the whole person, their individual goals, the complexities of their multiple conditions and their social needs is often preferred in contexts such as GR [38].

Everink et al. demonstrate that GR is not only medically beneficial, but also economically advantageous, particularly in terms of avoiding long-term care and repeated hospital stays. It is therefore recommended to consider GR as part of an integrated care pathway for older people [39]. The central criteria for rehabilitation indication should be functional status, level of independence, and potential need for care, rather than the presence of individual medical diagnoses [40, 41].

GR remains predominantly inpatient in the DACH region despite evidence suggesting comparable or even superior outcomes in outpatient rehabilitation [42, 43]. Results from the survey show that a wide range of outpatient or mobile services for older patients in the DACH region are designated as “rehabilitation or GR”. However, going into depth, it became clear that those initiatives frequently involve only one health professional group and therefore do not follow the quality criteria and standards as promoted by EuGMS [8]. This approach does not constitute rehabilitation per se, but rather may be seen as a single therapeutic measure aiming at ameliorating specific health problems. GR is invariably multidisciplinary in nature and is predicated on the CGA at the participation level, in accordance with the biopsychosocial model of the ICF [6, 41]. This is a lacking standard to meet future needs, when such approaches should be structurally and financially integrated into routine care [44]. However, ambulatory services are not universally available in the DACH region with GR missing phases 3 and 4 in all countries. This implies a clear gap when considering integrated care for older people as a “care standard” as promoted by WHO for sustainable rehabilitation outcomes [45].

It is evident that the team structures for rehabilitation are similar in all three countries. The core team in Germany and Switzerland is, in principle, also present in Austria’s disease-oriented rehabilitation centers. However, in Austria, the rehabilitation teams do not follow a structured CGA in phase 2 although CGA is designed to manage complex needs by coordinating multidimensional perspectives through an interdisciplinary treatment team [46, 47]. Moreover, in Austria, no mandatory geriatric qualifications exist for rehabilitation physicians, nurses or therapists. In contrast to the structured training programs available in Germany and Switzerland, Austrian qualifications are contingent on individual initiative. Against this background, a binding framework for all professional groups would be desirable [48]. In view of the general staff shortage, specific, comprehensive, and interesting training in geriatrics could be an incentive for all medical, therapeutic, and nursing professions to apply for work in a geriatric facility. This could help ensure that the very demanding work in geriatrics is seen as an opportunity to gain a comprehensive perspective on the care of older people, which encompasses not only a single problem but also takes into account multimorbidity and frailty. The mandatory integration of physiotherapy, occupational therapy, speech therapy, nutrition, and (neuro)psychology, in both individual and group settings, is widely recognized as an essential component of comprehensive care. It is important to note that this addresses the key clinical needs of geriatric patients [49–52]. Regular inter- and multidisciplinary team meetings, as conducted in practice, represent an indispensable element of the overall rehabilitation process [53, 54].

Another major difference between the three countries is the time of intervention offered to GR patients in the DACH region. In Germany, therapy minutes per week are only mandated for phase 1, at an average of 600 min (ranging from 300 to 900). In Switzerland, such a requirement only applies to phase 2, with a minimum of 300 min. According to the EuGMS document [8] “the therapeutic intensity has to be tailored to the need of the individual patient and often needs to accommodate the reduced capabilities of geriatric rehabilitation patients”. Given the fact that there is no literature cited in the European Consensus Statement [8], the content seems to be strongly based on expert consensus. Our results highlight the urgent need for action and further research on this topic as this remarkable finding bears the potential to impact patients and systems. One possible reason for this lack of evidence is the challenge posed by the heterogeneity of older multimorbid patients with functional deficits when using traditional research designs. However, this gap could be addressed by using AI-driven real-life data analysis or by applying a comparative effectiveness research design [55].

In order to meet future requirements, digital transformation of GR may drive developments around many of the topics raised in this publication. The utilization of AI in the field of GR has already attained an international scale and is undergoing rapid development [56, 57]. AI, mobile applications, health sensors, assistive robots, daily living support technologies, virtual reality, and exergames are only a few of the innovations, which need to be taken into account as driving hubs for GR [58, 59].

It could easily be argued that all of the findings published in this work could form the basis for future collaborative projects among rehabilitation experts in Europe. A detailed evaluation of multimorbidity and frailty in an integrated approach, from primary care to acute hospital and rehabilitation in all phases will be an essential cornerstone to address demographic shifts and related care delivery needs for European citizens. This paradigm shift will be essential also for funding bodies, facilitating appropriate referral decisions, and delivering person-centered GR [36].

### Strengths and limitations

This study demonstrates several methodological strengths. The survey was methodically designed following a systematic, evidence-based approach, aligning with the European Consensus Statement. Prior to dissemination, content validity and clarity were assured through the process of pretesting, which was conducted by experts in the field. Cross-national collaboration facilitated a structured comparison of varying health systems. The integration of descriptive and qualitative analysis in accordance with SRQR standards resulted in enhanced transparency and depth of interpretation.

However, it is important to acknowledge the inherent limitations of this approach. The exclusive involvement of a select and small group of experts along with the limited regional distribution may compromise the representativeness of the study. The data coverage was revealed to be inconsistent, as the Austrian experts could only describe phase 1 GR. Consequently, the later rehabilitation phases (3 and 4) could not be fully recorded in all three countries. Furthermore, the utilization of self-reported data from designated experts may introduce subjective biases. Despite the differences in terminology and care structures being clarified, it is possible that these factors may have influenced the interpretation between countries. Despite these limitations, the study provides valuable comparative insights and highlights structural gaps and development needs in GR in the DACH region.

## Conclusion

GR is a continuous, interprofessional, and person-centered process bridging all phases of rehabilitative care. This current state analysis highlights structural differences within the DACH region, particularly the absence of a dedicated phase 2 GR in Austria, the lack of mandatory professional qualifications, and the limited implementation of digital health solutions in the three countries. In summary, the recommendations of the European Consensus Statement on GR are largely implemented in the DACH region although there are country-specific differences. Future considerations for GR should include the development of standardized quality criteria and the integration of innovative models, particularly in mobile and home-based settings, to ensure long-term effectiveness, evidence-based quality, and sustainability. Further research is needed to identify areas for action and best practices for policymakers, grounded in robust empirical evidence and oriented toward optimizing geriatric care across settings [60].

## Supplementary Information

Below is the link to the electronic supplementary material.

Supplementary file1 (PDF 42 KB)

## Data Availability

The survey data are available from the authors upon reasonable request.
