# Supplementary material for: Geriatric rehabilitation in Germany, Austria, and Switzerland (DACH region): a current state analysis
Source: Eur Geriatr Med. 2026 Mar 29;17(3):1535–43. doi: 10.1007/s41999-026-01461-7 (PMC13309369; doi:10.1007/s41999-026-01461-7)
Supplement: Supplementary file 1 — Supplementary file1 (PDF 42 KB) [file 41999_2026_1461_MOESM1_ESM.pdf]

Additional File 1: Geriatric Rehabilitation in Germany, Austria, and Switzerland (DACH Region): A Current State Analysis

**Part 1 - STRUCTURE OF GERIATRIC REHABILITATION (GR)**		
**Phases of rehabilitation and terminology**		
1.	1.1.	Does the phase classification (phases 1 to 4) of rehabilitation according to the WHO correspond to the classification in your country?
	1.2.	*If no, please provide classification and description*
2.	2.1.	Is the term GR used for phase 1?
	2.2.	Is the term GR used for phase 2?
	2.3.	Is the term GR used for phase 3?
	2.4.	Is the term GR used for phase 4?
3.	3.1.	Does the definition of GR from the European Consensus Statement correspond to the accepted definition in your country?
	3.2.	*Justification or differing definition*
4.	4.1.	Does the definition of a geriatric patient from the European Consensus Statement correspond to the accepted definition in your country?
	4.2.	*Justification or differing definition*
**Resources and structures**		
5.	5.1.	Is geriatric prehabilitation available in your country?
	5.2.	Is acute geriatric/remobilisation phase 1 available in your country?
	5.3.	Is geriatric post-acute rehabilitation phase 2 available in your country?
	5.4.	Is GR phase 3 available in your country?
	5.5.	Is GR phase 4 available in your country?
6.	6.1.	Is Phase 1 GR in your country indication-specific?
	6.2.	Is Phase 1 GR cross-indication in your country?
	6.3.	*Comment*
7.	7.1.	Is Phase 2 GR indication-specific in your country?
	7.2.	Is Phase 2 GR cross-indication in your country?
	7.3.	Is Phase 2 GR available on an outpatient basis in your country?
	7.4.	*Comment*
	7.5.	Is Phase 2 GR available in your country?
	7.6.	*Comment*
	7.7.	Other (specify)

Team structure		
8.	8.1.	Does the team structure from the European Consensus Statement correspond to the structures in your country?
	8.2.	*Reason/comment*
9.		Which professional groups are working in phase 1:
	9.1.	Physicians
	9.2.	Physicians with geriatric qualification (e.g. Geriatricians)
	9.3.	Physical therapists
	9.4.	Occupational therapists
	9.5.	Psychologists
	9.6.	Neuropsychologists
	9.7.	Speech therapists
	9.8.	Dietitians/nutritional counseling
	9.9.	Massage therapists
	9.10.	Medical-technical assistants
	9.11.	Sports scientists
	9.12.	Pharmacists
	9.13.	Nurses
	9.14.	Wound managers
	9.15.	Incontinence advisors
	9.16.	Case and care managers
	9.17.	Social workers
	9.18.	Music therapists
	9.19.	Other
	9.20.	If "Other", please specify which one
10.		Which professional groups are working in phase 2:
	10.1.	Physicians
	10.2.	Physicians with geriatric qualification (e.g. Geriatricians)
	10.3.	Physical therapists
	10.4.	Occupational therapists
	10.5.	Psychologists
	10.6.	Neuropsychologists
	10.7.	Speech therapists
	10.8.	Dietitians/nutritional counseling
	10.9.	Massage therapists
	10.10.	Medical-technical assistants
	10.11.	Sports scientists

	10.12.	Pharmacists
	10.13.	Nurses
	10.14.	Wound managers
	10.15.	Incontinence advisors
	10.16.	Case and care managers
	10.17.	Social workers
	10.18.	Music therapists
	10.19.	Other
	10.20.	If "Other", please specify which one
**Standards, guidelines, directives**		
11.	11.1.	Which national/international standards/guidelines/directives are used for GR in your country?
12.	12.1.	Please provide a description from your country's perspective if the rationale/limits deviate from the European Consensus Statement or should be supplemented (e.g. minimum standards, relevant guidelines, admission standards or exclusion criteria, S3 guideline for geriatric assessment, manual for medical examiners, ST-Reha Catalog 3.0, CHOP Catalog Switzerland)
**Documentation/quality assurance systems, monitoring systems**		
13.	13.1.	QS Reha
	13.2.	ISO standard
	13.3.	Other quality management systems
14.	14.1.	Are there documentation systems that span multiple clinics?
15.	15.1.	Are there any cross-clinic control systems?
16.	16.1.	Please provide a description from your country's perspective if the rationale/limits of a systematic evaluation system deviates from the European Consensus Statement or should be supplemented (e.g. comprehensive documentation, quality, and control systems)
**Facilitators / barriers**		
17.	17.1.	Existing staff shortage
	17.2.	*[Rank 1]*
	17.3.	*[Rank 2]*
	17.4.	*[Rank 3]*
	17.5.	*[Rank 4]*

18.	18.1.	Insufficient qualification/training of personnel
	18.2.	*[Rank 1]*
	18.3.	*[Rank 2]*
	18.4.	*[Rank 3]*
	18.5.	*[Rank 4]*
19.	19.1.	Excessive workload for staff
	19.2.	*[Rank 1]*
	19.3.	*[Rank 2]*
	19.4.	*[Rank 3]*
	19.5.	*[Rank 4]*
20.	20.1.	Lack of further home care/support
	20.2.	Lack of standardised assessment tools
	20.3.	Increased cost pressure
	20.4.	Limited bed capacity
	20.5.	Reimbursement models inadequate (daily rates, fixed rates, etc.)
	20.6.	Inadequate legal regulations for GR
	20.7.	Other (specify)
**Part 2 - THE GERIATRIC REHABILITATION PROCESS**		
**Inclusion and exclusion criteria**		
21.	21.1.	Are there clear inclusion/exclusion criteria for phase 1 GR?
	21.2.	Are there clear inclusion/exclusion criteria for phase 2 GR?
22.	22.1.	Is the (optimally possible) assignment for phase 1 by (social insurance) bodies dependent on the frailty scale or similar assessments (the right patient in the right facility at the right time)?
	22.2.	Is the (optimally possible) assignment for phase 2 by (social insurance) bodies dependent on the frailty scale or similar assessments (the right patient in the right facility at the right time)?
	22.3.	Which instruments/scales are used in your country?
**GR approval**		
23.		Who approves rehabilitation for phase 1 in your country?
	23.1.	(Social) health insurance
	23.2.	(Social) pension insurance
	23.3.	Private insurance provider, other
24.		Who approves rehabilitation for phase 2 in your country?
	24.1.	(Social) health insurance
	24.2.	(Social) pension insurance
	24.3.	Private insurance provider, other
25.	25.1.	What rehabilitation duration is approved by your (social insurance) providers in phase 1?
	25.2.	What rehabilitation duration is approved by your (social insurance) providers in phase 2?
	25.3.	*If Other please explain in detail*

Facilites and staffing		
26.	26.1.	Are there nursing/bed wards with monitoring capabilities in the geriatric post-acute rehabilitation phase 2?
	26.2.	Are there residential and therapy rooms in phase 2 of geriatric post-acute rehabilitation that are specially equipped to meet the needs of geriatric patients?
27.	27.1.	Is there at least one physician in Phase 1 with specialist qualification in geriatrics, additional education in geriatrics, a medical diploma in geriatrics, or equivalent qualification?
	27.2.	Is there at least one physician in Phase 2 with specialist qualification in geriatrics, additional education in geriatrics, a medical diploma in geriatrics, or equivalent qualification?
28.	28.1.	Is there at least one employed physician or consulting physician for neurology/psychiatry and/or orthopedics in GR facilities during phase 1?
	28.2.	Is there at least one employed physician or consulting physician for neurology/psychiatry and/or orthopedics in GR facilities during phase 2?
29.	29.1.	Does a pharmacist participate in visits during phase 1?
	29.2.	Does a pharmacist participate in visits during phase 2?
30.	30.1.	Is there regular discharge management (case and care management) in Phase 1 GR in your country's facilities?
	30.2.	Is there regular discharge management (case and care management) in Phase 2 GR in your country's facilities?
	30.3.	Does the nursing staff carry out case and care management?
	30.4.	Do social workers carry out case and care management?
	30.5.	Which other professional groups carry out case and care management?
**ICF orientation**		
31.	31.1.	Is there participation-oriented rehabilitation based on the ICF's biopsychosocial model in phase 1 GR in your country?
	31.2.	Is there participation-oriented rehabilitation based on the ICF's biopsychosocial model in phase 2 GR in your country?
32.	32.1.	Is it mandatory to perform a comprehensive geriatric assessment upon admission and discharge in phase 1 GR?
	32.2.	Is it mandatory to perform a comprehensive geriatric assessment upon admission and discharge in phase 2 GR?
33.		How are rehabilitation goals formulated in phase 1 GR?
	33.1.	At the functional level according to the ICF
	33.2.	At the activity level according to the ICF
	33.3.	At the participation level according to the ICF
	33.4.	Other (specify)
34.		How are rehabilitation goals formulated in phase 2 GR?
	34.1.	At the functional level according to the ICF
	34.2.	At the activity level according to the ICF
	34.3.	At the participation level according to the ICF
	34.4.	Other (specify)
35.	35.1.	Are personalised rehabilitation goals formulated in the participation level according to the ICF model in acute geriatric care/remobilisation phase 1?
	35.2.	Are personalised rehabilitation goals formulated at the participation level according to the ICF model in geriatric post-acute rehabilitation phase 2?
36.	36.1.	Does goal setting take place in phase 1 with the involvement of patients (“shared decision making”)?
	36.2.	Does the target agreement take place in phase 2 with the involvement of the patients (“shared decision making”)?
37.	37.1.	Are family caregivers involved in the target agreement in phase 1?
	37.2.	Are family caregivers involved in the target agreement in phase 2?
38.	38.1.	Please describe the rehabilitation process from your country's perspective if it differs from the European Consensus Statement

Therapeutic specifications		
39.	39.1.	How is therapy prescribed in acute geriatric care/remobilisation phase 1?
	39.2.	How is therapy prescribed in phase 2 of geriatric post-acute rehabilitation?
40.	40.1.	Are interdisciplinary team meetings mandatory in Phase 1 GR in your country?
	40.2.	Are interdisciplinary team meetings mandatory in Phase 2 GR in your country?
41.	41.1.	Are there minimum therapy minutes that a patient must receive during their stay in Phase 1? If so, please specify the minimum therapy minutes per stay in numbers.
	41.2.	*Comment*
	41.3.	Are there minimum therapy minutes that a patient must receive during their stay in Phase 2? If so, please specify the minimum therapy minutes per stay in numbers.
	41.4.	*Comment*
	41.5.	Are there minimum therapy minutes that a patient must receive in a calendar week in Phase 1? If so, please specify the minimum therapy minutes per calendar week in numbers.
	41.6.	*Comment*
	41.7.	Are there minimum therapy minutes that a patient must receive in a calendar week in Phase 2? If so, please specify the minimum therapy minutes per calendar week in numbers.
	41.8.	*Comment*
42.		In which occupational groups are individual (1:1) therapies mandatory in phase 1?
	42.1.	Physical therapists
	42.2.	Occupational therapists
	42.3.	Speech therapists
	42.4.	Dietitians/nutritional counseling
	42.5.	Psychologists
	42.6.	No individual therapies are mandatory
	42.7.	Other (specify)
43.		In which occupational groups are individual (1:1) therapies mandatory in phase 2?
	43.1.	Physical therapists
	43.2.	Occupational therapists
	43.3.	Speech therapists
	43.4.	Dietitians/nutritional counseling
	43.5.	Psychologists
	43.6.	No individual therapies are mandatory
	43.7.	Other (specify)
44.	44.1.	What are the typical group sizes for group therapy in phase 1?
	44.2.	What are the typical group sizes for group therapy in phase 2?
	44.3.	*Explanation (other group sizes)*

**Diagnostics, assessments**		
45.		What type of diagnostics can generally be performed in phase 2 GR facilities?
	45.1.	Laboratory diagnostics
	45.2.	Medical diagnostics, e.g., ultrasound
	45.3.	X-ray diagnostics
	45.4.	Other (specify)
46.		What mandatory assessments are undertaken?
	46.1.	Malnutrition
	46.2.	Dysphagia
	46.3.	Fall risk
	46.4.	Other (specify)
**Outcome measurements**		
47.	47.1.	Which outcome measurements are used in phase 1 upon admission? (e.g., Barthel Index, EQ-5D, PHQ-4, 6MGT)
	47.2.	Which outcome measurements are used in phase 1 upon discharge? (e.g., Barthel Index, EQ-5D, PHQ-4, 6MGT)
	47.3.	Which outcome measurements are used in phase 1 upon admission and discharge? (e.g., Barthel Index, EQ-5D, PHQ-4, 6MGT)
48.	48.1.	Which outcome measurements are used in phase 2 upon admission? (e.g., Barthel Index, EQ-5D, PHQ-4, 6MGT)
	48.2.	Which outcome measurements are used in phase 2 upon discharge? (e.g., Barthel Index, EQ-5D, PHQ-4, 6MGT)
	48.3.	Which outcome measurements are used in phase 2 upon admission and discharge? (e.g., Barthel Index, EQ-5D, PHQ-4, 6MGT)
49.	49.1.	Are these outcome measurements used in phase 1 in accordance with the S3 assessment guideline in your country?
	49.2.	Are these outcome measurements used in phase 2 in accordance with the S3 assessment guideline in your country?
**Ad Part 1 - ADDITIONAL STANDARDS**		
**Education standards**		
50.		What types of training/education are available in the medical field?
	50.1.	Specialists in geriatrics
	50.2.	Additional training (qualification) in geriatrics
	50.3.	Diploma in Geriatrics
	50.4.	Other (specify)
	51.1.	Can you provide information on the scope of GR specific education in other professional groups?
	51.2.	Which professional group receives GR specific training?
	51.3.	Physical therapists
	51.4.	Occupational therapists
	51.5.	Psychologists
	51.6.	Neuropsychologists
	51.7.	Speech therapists
	51.8.	Dietitians/nutritional counseling
	51.9.	Massage therapists

51.	51.10.	Medical-technical assistants
	51.11.	Sports scientists
	51.12.	Pharmacists
	51.13.	Nurses
	51.14.	Wound managers
	51.15.	Incontinence advisors
	51.16.	Case and care managers
	51.17.	Social workers
	51.18.	Music therapists
	51.19.	Other (specify)
52.	52.1.	Please describe GR training from the perspective of your country if it differs from or should be supplemented by the European Consensus Statement (e.g. minimum training standards for professional groups, additional training)
**Part 3 - STRATEGIES FOR DIGITAL DEVELOPMENT**		
**Planned/currently implemented strategies**		
53.		Which of the following development strategies are planned/being implemented in your country?
	53.1.	Home-based/mobile GR
	53.2.	Telerehabilitation
	53.3.	eHealth-applications
	53.4.	Other (specify)
54.	54.1.	Please describe the strategies for further developing GR from your country's perspective if these differ from or should supplement the European Consensus Statement
55.	55.1.	Is eHealth already routinely used in Phase 1 in your country?
	55.2.	Is eHealth already routinely used in Phase 2 in your country?
**Use of eHealth**		
56.		Which eHealth applications are used?
	56.1.	Assistive robotics
	56.2.	Ambient Assisted Living solutions
	56.3.	Mobile applications
	56.4.	Virtual reality
	56.5.	Health Sensors
	56.6.	Exergames
	56.7.	Artificial Intelligence
	56.8.	Unknown
	56.9.	Other (specify)
57.	57.1.	Are there any plans for eHealth applications in GR in your country in the future?

Use of telerehabilitation		
58.	58.1.	Is telerehabilitation routinely used in GR in your country?
	58.2.	Is telerehabilitation carried out exclusively via video communication (or is telerehabilitation accompanied by therapists or the rehabilitation team on site (e.g. at home))?
	58.3.	Please describe the time frame (duration) and scope of therapy in which telerehabilitation is used.
59.	59.1.	Is telerehabilitation used in phase 1?
	59.2.	Is telerehabilitation used in phase 2?
60.		Which professional groups are involved?
	60.1.	Physicians
	60.2.	Physicians with geriatric qualification (e.g. Geriatricians)
	60.3.	Physical therapists
	60.4.	Occupational therapists
	60.5.	Psychologists
	60.6.	Neuropsychologists
	60.7.	Speech therapists
	60.8.	Dietitians/nutritional counseling
	60.9.	Massage therapists
	60.10.	Medical-technical assistants
	60.11.	Sports scientists
	60.12.	Pharmacists
	60.13.	Nurses
	60.14.	Wound managers
	60.15.	Incontinence advisors
	60.16.	Case and care managers
	60.17.	Social workers
	60.18.	Music therapists
	60.19.	Other (specify)
61.	61.1.	Is telerehabilitation planned for GR in your country in the future?
